# Triaqua[2,2′-(propane-1,3-diyl)bis(5-carboxy-1*H*-imidazole-4-carboxylato-κ^2^ *N* ^3^,*O* ^4^)]calcium(II) tetrahydrate

**DOI:** 10.1107/S1600536812035544

**Published:** 2012-08-23

**Authors:** Ling-Zhi Du, Xia Li

**Affiliations:** a Department of Chemistry and Chemical Engineering, Henan University of Urban Construction, Pingdingshan, Henan, 467044, People’s Republic of China

## Abstract

In the title compound, [Ca(C_13_H_10_N_4_O_8_)(H_2_O)_3_]·4H_2_O, the Ca^II^ ion is hepta-coordinated by two N atoms and two O atoms from a tetra­dentate 1,3-bis-(1*H*-imidazole-4,5-dicarb­oxy­l­ate) propane dianion and three water O atoms, giving a distorted penta­gonal–bipyramidal coordination environment. The Ca—O bond lengths are in the range 2.354 (3)–2.453 (2) Å, while the Ca—N bond lengths are in the range 2.523 (2)–2.548 (2) Å. An intra­molecular O—H⋯O hydrogen bond between the carb­oxy and carboxyl­ate groups stabilizes the mol­ecular configuration. A three-dimensional network of N—H⋯O and O—H⋯O hydrogen bonds help to stabilize the crystal packing.

## Related literature

For complexes based on 4,5-imidazole­dicarb­oxy­lic acid, see: Zhu *et al.* (2010 ▶); Lu *et al.* (2010 ▶). For complexes based on 2-methyl-1*H*-imidazole-4,5-dicarb­oxy­lic acid, see: Song *et al.* (2010 ▶). For complexes based on 2-ethyl-1*H*-imidazole-4,5-dicarb­oxy­lic acid, see: Zhang *et al.* (2010 ▶); Wang *et al.* (2008 ▶). For complexes based on 2-propyl-1*H*-imidazole-4,5-dicarb­oxy­lic acid, see: Feng *et al.* (2010 ▶); Liu *et al.* (2010 ▶). For complexes based on 2-(hy­droxy­meth­yl)-1*H*-imidazole-4,5-dicarb­oxy­lic acid, see: Zheng *et al.* (2011 ▶). For complexes based on 2-phenyl-1*H*-imidazole-4,5-dicarb­oxy­lic acid, see: Zhu *et al.* (2011 ▶). For complexes based on 2-pyridyl-1*H*-imidazole-4,5-dicarb­oxy­lic acid, see: Li *et al.* (2009 ▶, 2010 ▶).
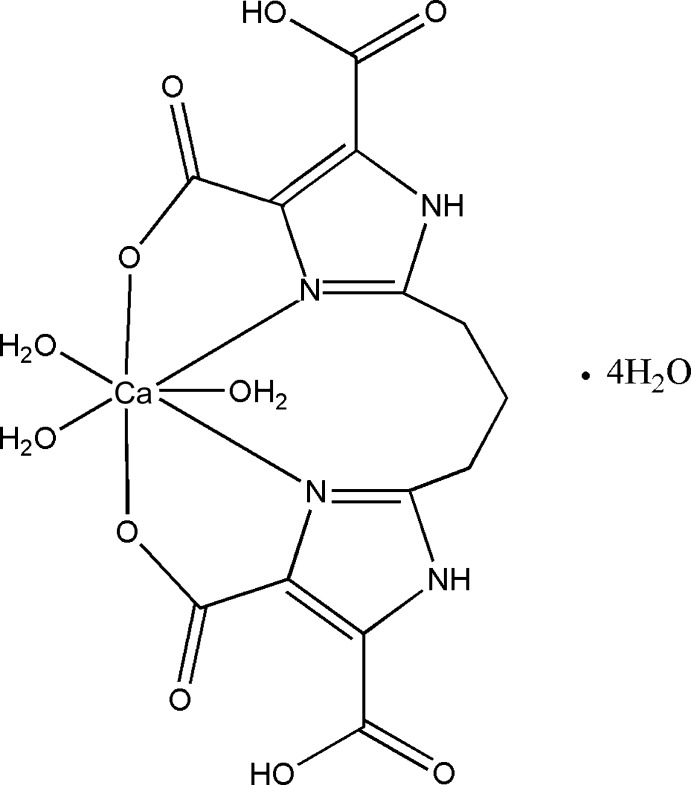


## Experimental

### 

#### Crystal data

[Ca(C_13_H_10_N_4_O_8_)(H_2_O_3_)_3_]·4H_2_O
*M* *_r_* = 516.44
Triclinic, 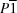

*a* = 6.7794 (12) Å
*b* = 12.172 (2) Å
*c* = 13.718 (2) Å
α = 98.776 (2)°
β = 102.420 (2)°
γ = 90.444 (2)°
*V* = 1091.6 (3) Å^3^
*Z* = 2
Mo *K*α radiation
μ = 0.37 mm^−1^
*T* = 296 K
0.16 × 0.16 × 0.14 mm

#### Data collection

Bruker SMART CCD diffractometer
Absorption correction: multi-scan (*SADABS*; Sheldrick, 2001 ▶) *T*_min_ = 0.943, *T*_max_ = 0.950
8318 measured reflections
4031 independent reflections
2595 reflections with *I* > 2σ(*I*)
*R*_int_ = 0.040

#### Refinement

*R*[*F* ^2^ > 2σ(*F* ^2^)] = 0.048
*wR*(*F* ^2^) = 0.098
*S* = 1.01
4031 reflections
344 parameters
14 restraints
H atoms treated by a mixture of independent and constrained refinement
Δρ_max_ = 0.24 e Å^−3^
Δρ_min_ = −0.32 e Å^−3^

### 

Data collection: *SMART* (Bruker, 2001 ▶); cell refinement: *SAINT* (Bruker, 2001 ▶); data reduction: *SAINT*; program(s) used to solve structure: *SHELXS97* (Sheldrick, 2008 ▶); program(s) used to refine structure: *SHELXL97* (Sheldrick, 2008 ▶); molecular graphics: *SHELXTL* (Sheldrick, 2008 ▶); software used to prepare material for publication: *SHELXTL*.

## Supplementary Material

Crystal structure: contains datablock(s) I, global. DOI: 10.1107/S1600536812035544/ff2078sup1.cif

Structure factors: contains datablock(s) I. DOI: 10.1107/S1600536812035544/ff2078Isup2.hkl

Additional supplementary materials:  crystallographic information; 3D view; checkCIF report

## Figures and Tables

**Table 1:** Hydrogen-bond geometry (Å, °)

*D*—H⋯*A*	*D*—H	H⋯*A*	*D*⋯*A*	*D*—H⋯*A*
N4—H4⋯O13^i^	0.86	1.94	2.778 (4)	164
O10—H3*W*⋯O5^ii^	0.86 (1)	2.03 (2)	2.839 (3)	159 (4)
O10—H4*W*⋯O1^iii^	0.86 (1)	1.91 (1)	2.768 (3)	174 (3)
O9—H2*W*⋯O5^iv^	0.86 (1)	1.93 (1)	2.784 (3)	172 (4)
O9—H1*W*⋯O12^v^	0.86 (1)	1.97 (1)	2.829 (4)	174 (5)
O14—H11*W*⋯O12^vi^	0.86 (1)	2.17 (2)	2.957 (4)	152 (4)
O15—H13*W*⋯O2^vi^	0.86 (1)	1.93 (2)	2.752 (3)	159 (4)
O15—H14*W*⋯O8^vii^	0.86 (1)	1.97 (1)	2.824 (3)	179 (4)
O13—H9*W*⋯O15^vi^	0.86 (1)	2.13 (2)	2.943 (4)	158 (4)
O13—H10*W*⋯O14^viii^	0.86 (1)	2.00 (1)	2.851 (4)	173 (5)
O11—H6*W*⋯O6^ii^	0.86 (1)	1.94 (2)	2.770 (3)	161 (4)
O11—H5*W*⋯O4^vi^	0.86 (1)	1.90 (2)	2.717 (3)	160 (4)
O12—H7*W*⋯O9^iii^	0.86 (1)	2.03 (2)	2.851 (3)	160 (5)
N2—H2⋯O14	0.86	1.96	2.809 (3)	169
O14—H12*W*⋯O15	0.86 (1)	1.88 (1)	2.740 (4)	177 (5)
O6—H6⋯O7	0.82	1.64	2.462 (3)	175
O3—H3⋯O2	0.82	1.67	2.487 (3)	176

Symmetry codes: (i) 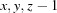; (ii) 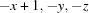; (iii) 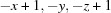; (iv) 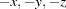; (v) 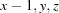; (vi) 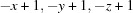; (vii) 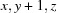; (viii) 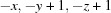.

**Fig. 1.**
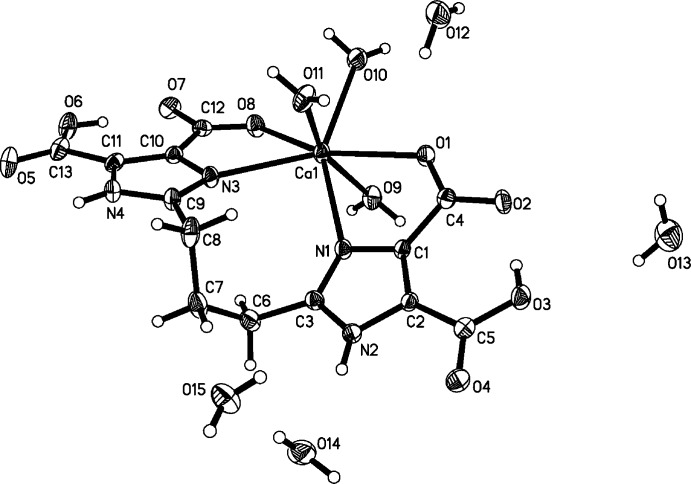
The molecular structure of the title compound, showing the atomic numbering and 30% probability displacement ellipsoids.

## supplementary crystallographic information

### Comment

Aromatic polycarboxylates, especially the N-heterocyclic carboxylates, are excellent candidates for preparing novel metal-organic frameworks, because of their versatile coordination modes and their ability to act as hydrogen-bonding donors and acceptors. For example, 4,5-imidazoledicarboxylate acid (Zhu *et al.*, 2010; Lu *et al.*, 2010), a planar rigid N-heterocyclic dicarboxylate acid, has been widely used to synthesize various coordination polymers because it has very flexible coordination modes, which derives from both imidazole and carboxylate functionality. Recently, in order to inherit the outstanding coordination properties of 4,5-imidazoledicarboxylic group, many 2-position substituent derivatives, such as 2-methyl-1*H*-imidazole-4,5-dicarboxylic acid (Song *et al.*, 2010), 2-ethyl-1*H*-imidazole-4,5-dicarboxylic acid (Zhang *et al.*, 2010; Wang *et al.*, 2008), 2-propyl-1*H*-imidazole-4,5-dicarboxylic acid (Feng *et al.*, 2010; Liu *et al.*, 2010), 2-(hydroxymethyl)-1*H*-imidazole-4,5-dicarboxylic acid (Zheng *et al.*, 20111), 2-phenyl-1*H*-imidazole-4,5-dicarboxylic acid (Zhu *et al.*, 2011) and 2-pyridyl-1*H*-imidazole-4,5-dicarboxylic acid (Li *et al.*, 2009; Li *et al.*, 2010) have been designed and used construct various metal complexes. Here, we want to report a calcium(II) complex, Ca(C_13_H_10_O_4_)(H_2_O)_3_].4H_2_O, based on a new imidazole dicarboxylate ligand, 1,3-Bis-(1*H*-imidazole-4,5-dicarboxylate acid).

As shown in Fig. 1, the molecule of (I) is a discrete neutral monomer, in which the asymmetric unit comprises a Ca^II^ ion, one 1,3-Bis-(1*H*-imidazole-4,5-dicarboxylate) propane dianion, three coordinated water molecules and four free water molecules. The Ca^II^ ion is hepta-coordinated, showing a distorted pentagonal-bipyramidal coordination environment. The equatorial plane is defined by two nitrogen atoms (N1, N3) and two oxygen atoms (O1, O8) from a 1,3-Bis-(1*H*-imidazole-4,5-dicarboxylate) propane dianion and one water molecule (O10). The axial positions are occupied by two coordinated water molecules with the bond angle of O9—Ca1—O11 being 159.80 (9) °. The Ca—O bond distances are in the range of 2.354 (3) - 2.453 (2) Å, while the Ca—N bond distances are in the range of 2.523 (2) - 2.548 (2) Å. An intramolecular O—H···O hydrogen bond between the carboxy and carboxylate groups stabilizes the molecular configuration. A three-dimensional network of N—H···O and O—H···O hydrogen bonds help to stabilize the crystal packing.

### Experimental

A mixture of calcium chloride dihydrate (0.0146 g, 0.1 mmol), 1,3-Bis-(1*H*-imidazole-4,5-dicarboxylate acid) propane (0.0352 g, 0.1 mmol), pyridine (0.8 ml) and H_2_O (10 ml) was sealed into a Teflon-lined stainless autoclave and heated at 413 K for 3 days. The bomb was allowed to cool to room temperature gradually and colorless block crystals of (I) were obtained.

### Refinement

H atoms attached to N and O atoms were located in a difference Fourier maps and refined as riding in their as-found relative positions, with *U*_iso_(H) = 1.5*U*_eq_(O,*N*). Other H atoms were positioned geometrically with C—H = 0.93 and 0.97 Å for aromatic and methyl H, and constrained to ride on their parent atoms with *U*_iso_(H) = 1.2*U*_eq_(C).

### Crystal data

**Table:**

[Ca(C_13_H_10_N_4_O_8_)(H_2_O_3_)_3_]·4H_2_O	*Z* = 2
*M**_r_* = 516.44	*F*(000) = 540
Triclinic, *P*1	*D*_x_ = 1.571 Mg m^−^^3^
Hall symbol: -P 1	Mo *K*α radiation, λ = 0.71073 Å
*a* = 6.7794 (12) Å	Cell parameters from 1354 reflections
*b* = 12.172 (2) Å	θ = 2.5–23.1°
*c* = 13.718 (2) Å	µ = 0.37 mm^−^^1^
α = 98.776 (2)°	*T* = 296 K
β = 102.420 (2)°	Block, colourless
γ = 90.444 (2)°	0.16 × 0.16 × 0.14 mm
*V* = 1091.6 (3) Å^3^	

### Data collection

**Table:**

Bruker SMART CCD diffractometer	4031 independent reflections
Radiation source: X-ray tube	2595 reflections with *I* > 2σ(*I*)
Phi and omega scans monochromator	*R*_int_ = 0.040
φ and ω scans	θ_max_ = 25.5°, θ_min_ = 2.5°
Absorption correction: multi-scan (*SADABS*; Sheldrick, 2001)	*h* = −8→8
*T*_min_ = 0.943, *T*_max_ = 0.950	*k* = −14→14
8318 measured reflections	*l* = −16→16

### Refinement

**Table:**

Refinement on *F*^2^	Primary atom site location: structure-invariant direct methods
Least-squares matrix: full	Secondary atom site location: difference Fourier map
*R*[*F*^2^ > 2σ(*F*^2^)] = 0.048	Hydrogen site location: inferred from neighbouring sites
*wR*(*F*^2^) = 0.098	H atoms treated by a mixture of independent and constrained refinement
*S* = 1.01	*w* = 1/[σ^2^(*F*_o_^2^) + (0.0327*P*)^2^ + 0.2508*P*] where *P* = (*F*_o_^2^ + 2*F*_c_^2^)/3
4031 reflections	(Δ/σ)_max_ < 0.001
344 parameters	Δρ_max_ = 0.24 e Å^−^^3^
14 restraints	Δρ_min_ = −0.32 e Å^−^^3^

### Special details

**Table:**

Geometry. All e.s.d.'s (except the e.s.d. in the dihedral angle between two l.s. planes) are estimated using the full covariance matrix. The cell e.s.d.'s are taken into account individually in the estimation of e.s.d.'s in distances, angles and torsion angles; correlations between e.s.d.'s in cell parameters are only used when they are defined by crystal symmetry. An approximate (isotropic) treatment of cell e.s.d.'s is used for estimating e.s.d.'s involving l.s. planes.
Refinement. Refinement of *F*^2^ against ALL reflections. The weighted *R*-factor *wR* and goodness of fit *S* are based on *F*^2^, conventional *R*-factors *R* are based on *F*, with *F* set to zero for negative *F*^2^. The threshold expression of *F*^2^ > σ(*F*^2^) is used only for calculating *R*-factors(gt) *etc*. and is not relevant to the choice of reflections for refinement. *R*-factors based on *F*^2^ are statistically about twice as large as those based on *F*, and *R*- factors based on ALL data will be even larger.

### Fractional atomic coordinates and isotropic or equivalent isotropic displacement parameters (Å^2^)

**Table:**

	*x*	*y*	*z*	*U*_iso_*/*U*_eq_	
Ca1	0.34365 (10)	0.10418 (5)	0.30772 (5)	0.02902 (18)	
O1	0.4347 (3)	0.17772 (16)	0.48632 (15)	0.0339 (5)	
O2	0.4429 (3)	0.31736 (17)	0.61241 (15)	0.0404 (6)	
O3	0.3556 (3)	0.51623 (17)	0.63708 (15)	0.0381 (6)	
H3	0.3814	0.4502	0.6309	0.046*	
O4	0.2140 (3)	0.63567 (16)	0.53912 (16)	0.0375 (6)	
O5	0.2380 (4)	0.07270 (19)	−0.21218 (17)	0.0498 (7)	
O6	0.1955 (4)	−0.08158 (19)	−0.14977 (16)	0.0489 (7)	
H6	0.1955	−0.0984	−0.0941	0.059*	
O7	0.2100 (3)	−0.14045 (16)	0.01549 (16)	0.0406 (6)	
O8	0.2447 (3)	−0.05781 (16)	0.17484 (16)	0.0379 (6)	
O9	0.0695 (4)	0.03304 (19)	0.36754 (17)	0.0414 (6)	
O10	0.5458 (4)	−0.0399 (2)	0.36928 (19)	0.0464 (6)	
O11	0.6575 (4)	0.1900 (2)	0.31047 (19)	0.0432 (6)	
O12	0.9370 (5)	0.1553 (2)	0.5324 (2)	0.0604 (8)	
O13	0.3808 (5)	0.3595 (3)	0.8695 (2)	0.0670 (8)	
O14	−0.0383 (4)	0.6606 (2)	0.2953 (2)	0.0514 (7)	
O15	0.2804 (4)	0.7234 (2)	0.21968 (19)	0.0511 (7)	
N1	0.2148 (4)	0.29607 (19)	0.34785 (17)	0.0282 (6)	
N2	0.1366 (4)	0.47230 (19)	0.36829 (18)	0.0278 (6)	
H2	0.0902	0.5349	0.3542	0.033*	
N3	0.2951 (4)	0.14885 (19)	0.12881 (17)	0.0296 (6)	
N4	0.2984 (4)	0.1999 (2)	−0.01827 (18)	0.0322 (6)	
H4	0.3066	0.2417	−0.0626	0.039*	
C1	0.2844 (4)	0.3426 (2)	0.4475 (2)	0.0246 (7)	
C2	0.2362 (4)	0.4526 (2)	0.4611 (2)	0.0248 (7)	
C3	0.1233 (5)	0.3772 (2)	0.3025 (2)	0.0281 (7)	
C4	0.3947 (5)	0.2746 (2)	0.5201 (2)	0.0288 (7)	
C5	0.2698 (4)	0.5412 (2)	0.5491 (2)	0.0282 (7)	
C6	0.0236 (5)	0.3679 (3)	0.1933 (2)	0.0382 (9)	
H6A	−0.1009	0.4076	0.1872	0.046*	
H6B	−0.0116	0.2902	0.1661	0.046*	
C7	0.1548 (6)	0.4140 (3)	0.1298 (2)	0.0471 (10)	
H7A	0.1873	0.4922	0.1560	0.056*	
H7B	0.0775	0.4087	0.0609	0.056*	
C8	0.3519 (5)	0.3533 (2)	0.1291 (2)	0.0421 (9)	
H8A	0.4324	0.3909	0.0926	0.050*	
H8B	0.4284	0.3574	0.1981	0.050*	
C9	0.3172 (5)	0.2347 (2)	0.0816 (2)	0.0311 (8)	
C10	0.2618 (4)	0.0566 (2)	0.0547 (2)	0.0271 (7)	
C11	0.2642 (4)	0.0872 (2)	−0.0371 (2)	0.0300 (7)	
C12	0.2371 (4)	−0.0524 (2)	0.0845 (2)	0.0305 (7)	
C13	0.2322 (5)	0.0231 (3)	−0.1405 (3)	0.0376 (8)	
H5W	0.720 (5)	0.247 (2)	0.350 (2)	0.083 (15)*	
H6W	0.726 (5)	0.167 (3)	0.266 (2)	0.080 (15)*	
H4W	0.542 (5)	−0.083 (2)	0.4128 (19)	0.054 (12)*	
H3W	0.612 (5)	−0.067 (3)	0.325 (2)	0.070 (14)*	
H13W	0.370 (5)	0.728 (4)	0.276 (2)	0.106*	
H12W	0.062 (5)	0.683 (4)	0.273 (3)	0.106*	
H2W	−0.032 (4)	0.000 (3)	0.324 (3)	0.106*	
H14W	0.269 (7)	0.7895 (16)	0.205 (3)	0.106*	
H11W	−0.052 (7)	0.709 (3)	0.346 (2)	0.106*	
H9W	0.498 (3)	0.345 (4)	0.858 (4)	0.106*	
H7W	0.904 (7)	0.102 (3)	0.560 (3)	0.106*	
H10W	0.280 (5)	0.360 (4)	0.820 (2)	0.106*	
H1W	0.026 (6)	0.074 (3)	0.415 (2)	0.106*	
H8W	0.844 (5)	0.197 (3)	0.509 (3)	0.106*	

### Atomic displacement parameters (Å^2^)

**Table:**

	*U*^11^	*U*^22^	*U*^33^	*U*^12^	*U*^13^	*U*^23^
Ca1	0.0426 (4)	0.0223 (3)	0.0218 (4)	0.0014 (3)	0.0072 (3)	0.0021 (3)
O1	0.0529 (15)	0.0228 (11)	0.0259 (13)	0.0087 (10)	0.0080 (11)	0.0040 (9)
O2	0.0593 (16)	0.0390 (13)	0.0192 (12)	0.0103 (11)	0.0017 (11)	0.0028 (10)
O3	0.0533 (15)	0.0305 (12)	0.0266 (13)	0.0024 (11)	0.0052 (11)	−0.0029 (10)
O4	0.0464 (14)	0.0253 (12)	0.0377 (14)	0.0017 (11)	0.0076 (11)	−0.0020 (10)
O5	0.0657 (18)	0.0576 (16)	0.0262 (14)	−0.0151 (13)	0.0167 (12)	−0.0011 (12)
O6	0.0706 (18)	0.0461 (15)	0.0284 (14)	−0.0049 (13)	0.0158 (12)	−0.0062 (11)
O7	0.0509 (15)	0.0258 (12)	0.0396 (14)	0.0010 (11)	0.0072 (12)	−0.0082 (10)
O8	0.0527 (15)	0.0292 (12)	0.0305 (14)	0.0000 (11)	0.0054 (11)	0.0054 (10)
O9	0.0456 (16)	0.0444 (15)	0.0321 (15)	−0.0064 (12)	0.0074 (12)	0.0017 (11)
O10	0.0652 (18)	0.0437 (15)	0.0390 (16)	0.0190 (13)	0.0217 (14)	0.0182 (13)
O11	0.0504 (17)	0.0382 (14)	0.0371 (16)	−0.0096 (12)	0.0149 (13)	−0.0127 (12)
O12	0.087 (2)	0.0513 (18)	0.0465 (17)	0.0267 (15)	0.0146 (15)	0.0186 (13)
O13	0.075 (2)	0.075 (2)	0.058 (2)	−0.0009 (19)	0.0211 (17)	0.0238 (16)
O14	0.0622 (18)	0.0382 (15)	0.0601 (19)	0.0109 (14)	0.0211 (15)	0.0165 (12)
O15	0.0573 (18)	0.0431 (15)	0.0495 (17)	−0.0013 (14)	−0.0018 (13)	0.0165 (13)
N1	0.0368 (16)	0.0250 (13)	0.0210 (14)	0.0012 (12)	0.0037 (12)	0.0015 (11)
N2	0.0352 (16)	0.0204 (13)	0.0263 (15)	0.0020 (11)	0.0053 (12)	0.0015 (11)
N3	0.0404 (17)	0.0260 (14)	0.0195 (14)	−0.0014 (12)	0.0030 (12)	−0.0004 (11)
N4	0.0396 (17)	0.0356 (15)	0.0225 (15)	−0.0009 (13)	0.0082 (12)	0.0063 (12)
C1	0.0309 (18)	0.0224 (16)	0.0207 (17)	0.0006 (13)	0.0073 (14)	0.0015 (13)
C2	0.0263 (17)	0.0258 (16)	0.0215 (16)	0.0012 (13)	0.0061 (14)	0.0004 (13)
C3	0.0319 (19)	0.0247 (16)	0.0279 (18)	0.0013 (14)	0.0057 (15)	0.0057 (14)
C4	0.0320 (19)	0.0275 (17)	0.0275 (19)	−0.0011 (14)	0.0081 (15)	0.0045 (14)
C5	0.0250 (18)	0.0281 (18)	0.0306 (19)	−0.0040 (14)	0.0071 (15)	0.0005 (14)
C6	0.050 (2)	0.0294 (18)	0.0276 (19)	0.0061 (16)	−0.0030 (16)	−0.0017 (15)
C7	0.086 (3)	0.0274 (18)	0.0260 (19)	0.0087 (19)	0.0066 (19)	0.0060 (15)
C8	0.066 (3)	0.0328 (19)	0.0253 (19)	−0.0139 (18)	0.0116 (18)	−0.0024 (15)
C9	0.042 (2)	0.0301 (17)	0.0214 (18)	−0.0005 (15)	0.0074 (15)	0.0034 (14)
C10	0.0255 (17)	0.0289 (16)	0.0253 (18)	0.0015 (14)	0.0050 (14)	0.0001 (14)
C11	0.0282 (18)	0.0314 (18)	0.0276 (19)	−0.0001 (14)	0.0074 (15)	−0.0058 (14)
C12	0.0256 (18)	0.0288 (17)	0.034 (2)	0.0036 (14)	0.0026 (15)	0.0004 (15)
C13	0.035 (2)	0.046 (2)	0.030 (2)	−0.0069 (17)	0.0116 (16)	−0.0051 (17)

### Geometric parameters (Å, º)

**Table:**

Ca1—O11	2.354 (3)	O14—H11W	0.860 (10)
Ca1—O10	2.379 (3)	O15—H13W	0.864 (10)
Ca1—O9	2.393 (3)	O15—H14W	0.859 (10)
Ca1—O1	2.420 (2)	N1—C3	1.333 (3)
Ca1—O8	2.453 (2)	N1—C1	1.377 (3)
Ca1—N1	2.523 (2)	N2—C3	1.345 (3)
Ca1—N3	2.548 (2)	N2—C2	1.364 (3)
Ca1—H3W	2.78 (3)	N2—H2	0.8600
O1—C4	1.253 (3)	N3—C9	1.333 (4)
O2—C4	1.265 (3)	N3—C10	1.376 (3)
O3—C5	1.304 (3)	N4—C9	1.349 (3)
O3—H3	0.8200	N4—C11	1.366 (4)
O4—C5	1.232 (3)	N4—H4	0.8600
O5—C13	1.237 (4)	C1—C2	1.375 (4)
O6—C13	1.279 (4)	C1—C4	1.475 (4)
O6—H6	0.8200	C2—C5	1.467 (4)
O7—C12	1.301 (3)	C3—C6	1.492 (4)
O8—C12	1.242 (3)	C6—C7	1.529 (5)
O9—H2W	0.861 (10)	C6—H6A	0.9700
O9—H1W	0.859 (10)	C6—H6B	0.9700
O10—H4W	0.859 (10)	C7—C8	1.533 (5)
O10—H3W	0.855 (10)	C7—H7A	0.9700
O11—H5W	0.855 (10)	C7—H7B	0.9700
O11—H6W	0.858 (10)	C8—C9	1.486 (4)
O12—H7W	0.858 (10)	C8—H8A	0.9700
O12—H8W	0.852 (10)	C8—H8B	0.9700
O13—H9W	0.856 (10)	C10—C11	1.370 (4)
O13—H10W	0.856 (10)	C10—C12	1.465 (4)
O14—H12W	0.862 (10)	C11—C13	1.482 (4)
			
O11—Ca1—O10	83.73 (10)	C10—N3—Ca1	114.06 (19)
O11—Ca1—O9	159.80 (9)	C9—N4—C11	108.4 (2)
O10—Ca1—O9	89.24 (9)	C9—N4—H4	125.8
O11—Ca1—O1	81.93 (8)	C11—N4—H4	125.8
O10—Ca1—O1	79.49 (8)	C2—C1—N1	109.7 (3)
O9—Ca1—O1	78.17 (8)	C2—C1—C4	130.5 (3)
O11—Ca1—O8	114.31 (8)	N1—C1—C4	119.9 (2)
O10—Ca1—O8	75.75 (8)	N2—C2—C1	105.6 (2)
O9—Ca1—O8	81.96 (8)	N2—C2—C5	120.8 (3)
O1—Ca1—O8	148.28 (7)	C1—C2—C5	133.6 (3)
O11—Ca1—N1	87.84 (8)	N1—C3—N2	110.8 (3)
O10—Ca1—N1	146.79 (9)	N1—C3—C6	126.0 (3)
O9—Ca1—N1	87.76 (8)	N2—C3—C6	123.1 (3)
O1—Ca1—N1	67.53 (7)	O1—C4—O2	124.0 (3)
O8—Ca1—N1	136.25 (8)	O1—C4—C1	117.8 (3)
O11—Ca1—N3	77.65 (9)	O2—C4—C1	118.2 (3)
O10—Ca1—N3	124.49 (9)	O4—C5—O3	121.7 (3)
O9—Ca1—N3	121.47 (8)	O4—C5—C2	120.1 (3)
O1—Ca1—N3	145.79 (7)	O3—C5—C2	118.3 (3)
O8—Ca1—N3	65.91 (7)	C3—C6—C7	113.7 (3)
N1—Ca1—N3	84.44 (8)	C3—C6—H6A	108.8
O11—Ca1—H3W	76.2 (7)	C7—C6—H6A	108.8
O10—Ca1—H3W	16.9 (5)	C3—C6—H6B	108.8
O9—Ca1—H3W	101.5 (6)	C7—C6—H6B	108.8
O1—Ca1—H3W	93.4 (6)	H6A—C6—H6B	107.7
O8—Ca1—H3W	66.6 (7)	C6—C7—C8	113.6 (3)
N1—Ca1—H3W	156.9 (8)	C6—C7—H7A	108.9
N3—Ca1—H3W	107.8 (5)	C8—C7—H7A	108.9
C4—O1—Ca1	121.77 (19)	C6—C7—H7B	108.9
C5—O3—H3	109.5	C8—C7—H7B	108.9
C13—O6—H6	109.5	H7A—C7—H7B	107.7
C12—O8—Ca1	121.90 (19)	C9—C8—C7	112.8 (3)
Ca1—O9—H2W	118 (3)	C9—C8—H8A	109.0
Ca1—O9—H1W	119 (3)	C7—C8—H8A	109.0
H2W—O9—H1W	109 (4)	C9—C8—H8B	109.0
Ca1—O10—H4W	136 (2)	C7—C8—H8B	109.0
Ca1—O10—H3W	109 (3)	H8A—C8—H8B	107.8
H4W—O10—H3W	112 (4)	N3—C9—N4	110.5 (2)
Ca1—O11—H5W	130 (3)	N3—C9—C8	126.2 (3)
Ca1—O11—H6W	121 (3)	N4—C9—C8	123.3 (3)
H5W—O11—H6W	109 (4)	C11—C10—N3	110.0 (3)
H7W—O12—H8W	118 (5)	C11—C10—C12	131.7 (3)
H9W—O13—H10W	120 (5)	N3—C10—C12	118.3 (3)
H12W—O14—H11W	108 (4)	N4—C11—C10	105.5 (3)
H13W—O15—H14W	106 (4)	N4—C11—C13	122.0 (3)
C3—N1—C1	105.5 (2)	C10—C11—C13	132.5 (3)
C3—N1—Ca1	141.1 (2)	O8—C12—O7	122.1 (3)
C1—N1—Ca1	112.41 (17)	O8—C12—C10	119.0 (3)
C3—N2—C2	108.4 (2)	O7—C12—C10	118.9 (3)
C3—N2—H2	125.8	O5—C13—O6	124.0 (3)
C2—N2—H2	125.8	O5—C13—C11	119.3 (3)
C9—N3—C10	105.6 (2)	O6—C13—C11	116.7 (3)
C9—N3—Ca1	139.60 (19)		
			
O11—Ca1—O1—C4	86.6 (2)	Ca1—N1—C3—N2	−165.8 (2)
O10—Ca1—O1—C4	171.7 (2)	C1—N1—C3—C6	179.7 (3)
O9—Ca1—O1—C4	−96.9 (2)	Ca1—N1—C3—C6	12.4 (5)
O8—Ca1—O1—C4	−149.2 (2)	C2—N2—C3—N1	−1.6 (3)
N1—Ca1—O1—C4	−4.4 (2)	C2—N2—C3—C6	−179.9 (3)
N3—Ca1—O1—C4	33.0 (3)	Ca1—O1—C4—O2	−178.5 (2)
O11—Ca1—O8—C12	−54.5 (2)	Ca1—O1—C4—C1	1.6 (4)
O10—Ca1—O8—C12	−130.7 (2)	C2—C1—C4—O1	−173.7 (3)
O9—Ca1—O8—C12	138.0 (2)	N1—C1—C4—O1	5.3 (4)
O1—Ca1—O8—C12	−170.5 (2)	C2—C1—C4—O2	6.3 (5)
N1—Ca1—O8—C12	59.8 (3)	N1—C1—C4—O2	−174.7 (3)
N3—Ca1—O8—C12	8.2 (2)	N2—C2—C5—O4	−2.5 (4)
O11—Ca1—N1—C3	90.9 (3)	C1—C2—C5—O4	177.9 (3)
O10—Ca1—N1—C3	166.0 (3)	N2—C2—C5—O3	176.2 (3)
O9—Ca1—N1—C3	−108.8 (3)	C1—C2—C5—O3	−3.4 (5)
O1—Ca1—N1—C3	173.1 (3)	N1—C3—C6—C7	−99.3 (4)
O8—Ca1—N1—C3	−32.8 (4)	N2—C3—C6—C7	78.7 (4)
N3—Ca1—N1—C3	13.2 (3)	C3—C6—C7—C8	61.5 (3)
O11—Ca1—N1—C1	−75.75 (19)	C6—C7—C8—C9	63.7 (4)
O10—Ca1—N1—C1	−0.7 (3)	C10—N3—C9—N4	0.2 (3)
O9—Ca1—N1—C1	84.53 (19)	Ca1—N3—C9—N4	169.1 (2)
O1—Ca1—N1—C1	6.41 (18)	C10—N3—C9—C8	178.6 (3)
O8—Ca1—N1—C1	160.45 (17)	Ca1—N3—C9—C8	−12.6 (5)
N3—Ca1—N1—C1	−153.55 (19)	C11—N4—C9—N3	−0.4 (4)
O11—Ca1—N3—C9	−51.1 (3)	C11—N4—C9—C8	−178.8 (3)
O10—Ca1—N3—C9	−124.4 (3)	C7—C8—C9—N3	−89.7 (4)
O9—Ca1—N3—C9	121.9 (3)	C7—C8—C9—N4	88.5 (4)
O1—Ca1—N3—C9	3.7 (4)	C9—N3—C10—C11	0.0 (3)
O8—Ca1—N3—C9	−175.1 (3)	Ca1—N3—C10—C11	−172.1 (2)
N1—Ca1—N3—C9	37.9 (3)	C9—N3—C10—C12	178.3 (3)
O11—Ca1—N3—C10	117.1 (2)	Ca1—N3—C10—C12	6.3 (3)
O10—Ca1—N3—C10	43.8 (2)	C9—N4—C11—C10	0.4 (3)
O9—Ca1—N3—C10	−69.9 (2)	C9—N4—C11—C13	178.7 (3)
O1—Ca1—N3—C10	171.85 (18)	N3—C10—C11—N4	−0.3 (3)
O8—Ca1—N3—C10	−6.88 (19)	C12—C10—C11—N4	−178.3 (3)
N1—Ca1—N3—C10	−153.9 (2)	N3—C10—C11—C13	−178.3 (3)
C3—N1—C1—C2	−0.8 (3)	C12—C10—C11—C13	3.7 (6)
Ca1—N1—C1—C2	170.56 (19)	Ca1—O8—C12—O7	171.1 (2)
C3—N1—C1—C4	180.0 (3)	Ca1—O8—C12—C10	−8.2 (4)
Ca1—N1—C1—C4	−8.6 (3)	C11—C10—C12—O8	178.6 (3)
C3—N2—C2—C1	1.0 (3)	N3—C10—C12—O8	0.8 (4)
C3—N2—C2—C5	−178.7 (3)	C11—C10—C12—O7	−0.6 (5)
N1—C1—C2—N2	−0.1 (3)	N3—C10—C12—O7	−178.5 (3)
C4—C1—C2—N2	179.0 (3)	N4—C11—C13—O5	0.5 (5)
N1—C1—C2—C5	179.5 (3)	C10—C11—C13—O5	178.2 (3)
C4—C1—C2—C5	−1.4 (6)	N4—C11—C13—O6	−178.0 (3)
C1—N1—C3—N2	1.5 (3)	C10—C11—C13—O6	−0.3 (5)

### Hydrogen-bond geometry (Å, º)

**Table:**

*D*—H···*A*	*D*—H	H···*A*	*D*···*A*	*D*—H···*A*
N4—H4···O13^i^	0.86	1.94	2.778 (4)	164
O10—H3*W*···O5^ii^	0.86 (1)	2.03 (2)	2.839 (3)	159 (4)
O10—H4*W*···O1^iii^	0.86 (1)	1.91 (1)	2.768 (3)	174 (3)
O9—H2*W*···O5^iv^	0.86 (1)	1.93 (1)	2.784 (3)	172 (4)
O9—H1*W*···O12^v^	0.86 (1)	1.97 (1)	2.829 (4)	174 (5)
O14—H11*W*···O12^vi^	0.86 (1)	2.17 (2)	2.957 (4)	152 (4)
O15—H13*W*···O2^vi^	0.86 (1)	1.93 (2)	2.752 (3)	159 (4)
O15—H14*W*···O8^vii^	0.86 (1)	1.97 (1)	2.824 (3)	179 (4)
O13—H9*W*···O15^vi^	0.86 (1)	2.13 (2)	2.943 (4)	158 (4)
O13—H10*W*···O14^viii^	0.86 (1)	2.00 (1)	2.851 (4)	173 (5)
O11—H6*W*···O6^ii^	0.86 (1)	1.94 (2)	2.770 (3)	161 (4)
O11—H5*W*···O4^vi^	0.86 (1)	1.90 (2)	2.717 (3)	160 (4)
O12—H7*W*···O9^iii^	0.86 (1)	2.03 (2)	2.851 (3)	160 (5)
N2—H2···O14	0.86	1.96	2.809 (3)	169
O14—H12*W*···O15	0.86 (1)	1.88 (1)	2.740 (4)	177 (5)
O6—H6···O7	0.82	1.64	2.462 (3)	175
O3—H3···O2	0.82	1.67	2.487 (3)	176

Symmetry codes: (i) *x*, *y*, *z*−1; (ii) −*x*+1, −*y*, −*z*; (iii) −*x*+1, −*y*, −*z*+1; (iv) −*x*, −*y*, −*z*; (v) *x*−1, *y*, *z*; (vi) −*x*+1, −*y*+1, −*z*+1; (vii) *x*, *y*+1, *z*; (viii) −*x*, −*y*+1, −*z*+1.
